# Commissioning of the Tender X-ray Beamline at the High Energy Photon Source

**DOI:** 10.1107/S1600577525008446

**Published:** 2025-10-24

**Authors:** Yongyang Wang, Kun Tang, Shuhu Liu, Chenyan Ma, Xiaojuan Zhao, Dongmei Liu, Yidong Zhao, Lei Zheng

**Affiliations:** a Institute of High Energy Physics Chinese Academy of Sciences Beijing People’s Republic of China; b University of Chinese Academy of Sciences Beijing People’s Republic of China; Bhabha Atomic Research Centre, India

**Keywords:** TEX beamline, dynamic diagnostic tools, photon flux, energy resolution, diagnostic process

## Abstract

The Tender X-ray Beamline (TEX), using a bending magnet as a light source, is the first beamline of the High Energy Photon Source (HEPS) to undergo commissioning. It covers an energy range from 2.1 keV to 11 keV. Dynamic diagnostic tools have been installed and can measure the photon flux, energy resolution and position stability. By use of these tools, TEX was found to achieve a photon flux of up to 9 × 10^11^ photons s^−1^, with an energy resolution of 5904 @ 3203.6 eV and position stability lower than ±3 µm (horizontal) × 15 µm (vertical). In this paper, the diagnostic process of the beamline and its performance will be introduced in detail.

## Abstract 2

The High Energy Photon Source (HEPS) is the first fourth-generation synchrotron radiation source in China. In this paper, the diagnostic process of the Tender X-ray Beamline (TEX), the first bending magnet beamline of HEPS, and its performance are introduced in detail.

## 1. Introduction

The Tender X-ray Beamline (TEX) at the High Energy Photon Source (HEPS) in China (Jiao *et al.*, 2022 ▸; Jiao & Pan, 2022 ▸; Sun & Li, 2024 ▸) is specifically designed for X-ray absorption spectroscopy (XAS) (Grünert & Klementiev, 2020 ▸; Chantler *et al.*, 2024 ▸) in the energy range 2.1–11 keV. This energy range is particularly suitable for analyzing the *K*-edge absorption of elements with atomic number 15–31 and the *L*-edge absorption of elements with atomic number 38–71. These elemental analyses support energy and environmental research, including soil biogeochemistry, catalyst surface or interface mechanisms, and microbe–metal interaction mechanisms. Beyond environmental and energy applications, TEX can also be applied to biomedicine, chemistry and materials science. However, beamlines with energies as low as 2.1 keV are uncommon because of the stringent requirements of vacuum environments or 1 atm He environment in the chamber.

X-ray absorption fine structure (XAFS) spectroscopy experiments need precise energy scanning and a smooth flux spectrum at the sample position to ensure the accuracy of absorption spectroscopy measurements. The beamline adopts a bending magnet as the light source and employs a conventional collimation–monochromatizating–focusing optical design and layout. This optimized design enables good energy resolution and reliability performance, making the beamline a robust platform for synchrotron radiation studies.

## 2. Beamline description

**Figure 1:**
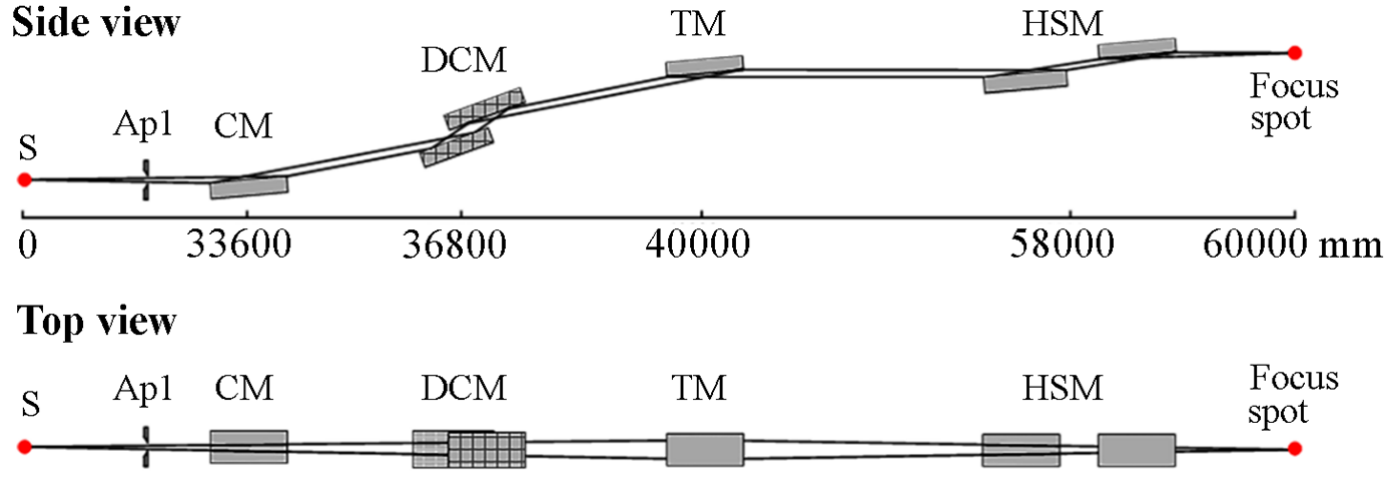
Schematic view of the TEX beamline, showing the collimating mirror (CM), double crystal monochromator (DCM), toroidal focusing mirror (TM) and harmonic suppression mirror (HSM).

**Figure 2:**
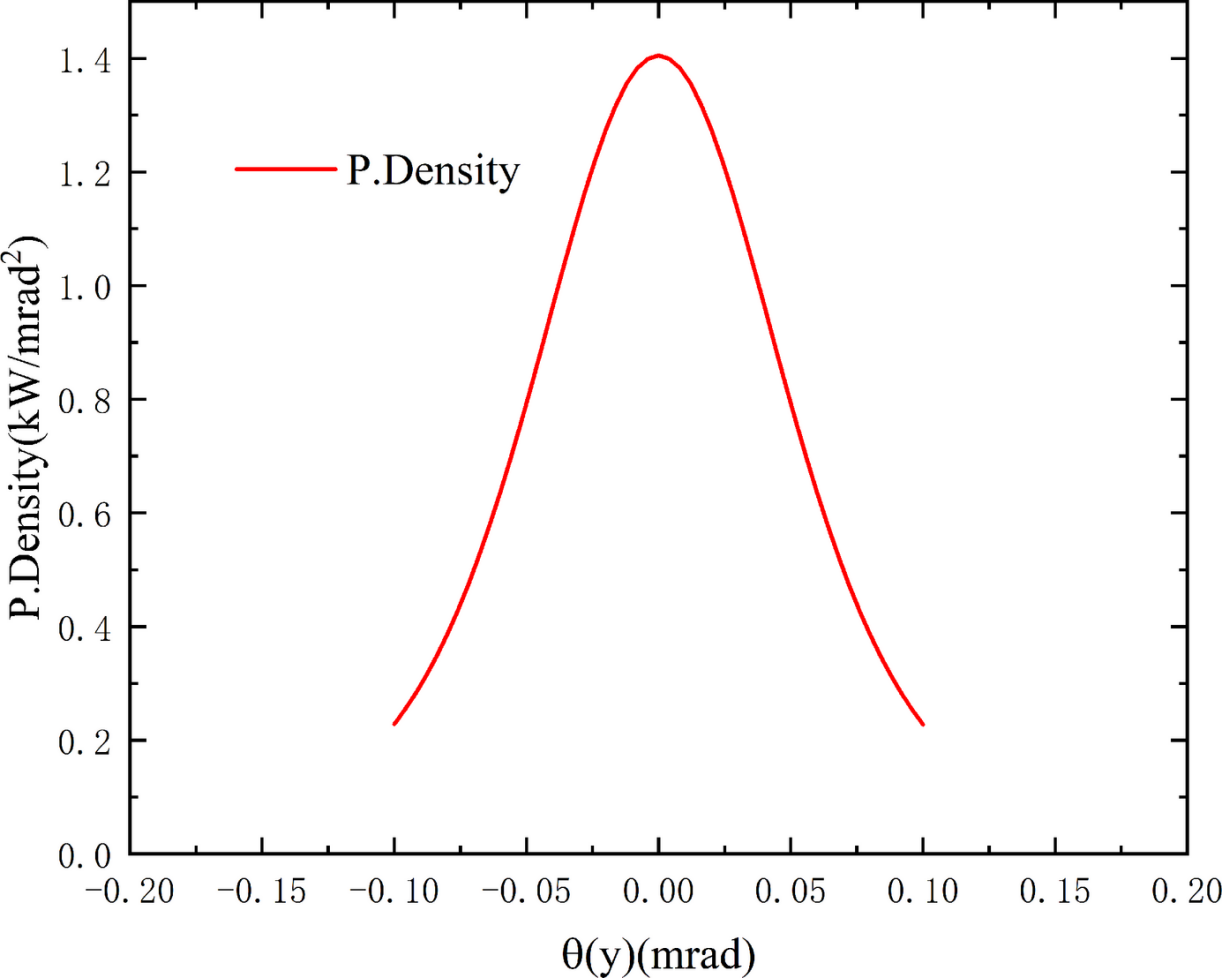
Angular power distribution in the vertical direction of the source.

The optical layout of TEX is given in Fig. 1 ▸. The source is a bending magnet with a field of 1 T and a critical energy of 23.9 keV. A series of fixed masks define an acceptance angle of 1.7 mrad × 0.2 mrad (H ×V) for the beamline. The bending magnet source has a total power of 1145 kW. Within an acceptance angle of 2 mrad × 1 mrad (H × V), 366 W is collected, while 279 W is collected within a narrower aperture of 1.7 mrad × 0.2 mrad (H × V). Angular power distribution in the vertical direction of the source is shown in Fig. 2 ▸. The overall design of the beamline includes a collimating mirror, Si (111) monochromator, focusing mirror and harmonic rejection mirrors.

The first optical component in the system is a vertically collimating mirror (CM) positioned at a distance of 33.6 m from the light source. Fabricated using a silicon substrate with a boron carbide/platinum (B_4_C/Pt) bilayer coating, this mirror has a grazing incidence angle of 7 mrad to the optical path. Downstream of the collimating mirror is a fixed-exit double crystal monochromator (DCM) (located at a distance of 36.8 m from the light source) with water cooling, which employs Si (111) crystals for energy dispersion in the vertical plane, enabling precise energy resolution control. The DCM was produced by KOHZU (Okui *et al.*, 2022 ▸). Its maximum speed is 1° s^−1^ (applicable to 4–11 keV), but the mechanical stability will not be stable enough at high angle conditions. For low energy (<4 keV), the operating speed needs to be reduced to 0.25° s^−1^ to ensure system stability. A toroidal focusing mirror (TM) with grazing incidence angle of 7 mrad is located at a distance of 40 m from the light source. It employs a focusing design with a horizontal compression ratio of 2:1 to minimize the aberration. It focuses the monochromated beam in both directions, ultimately forming an experimental focus at a distance of 60 m from the light source. Two meters upstream of the sample point is the harmonic suppression mirror (HSM), a system composed of two parallel plane mirrors. Each mirror contains three layers of coating (B_4_C, Ni and Pt) with working energy ranges corresponding to 2.1 keV–4.4 keV, 4.0 keV–7.8 keV and 7.5 keV–11 keV, and 7 mrad of the grazing incidence angle.

**Figure 3:**
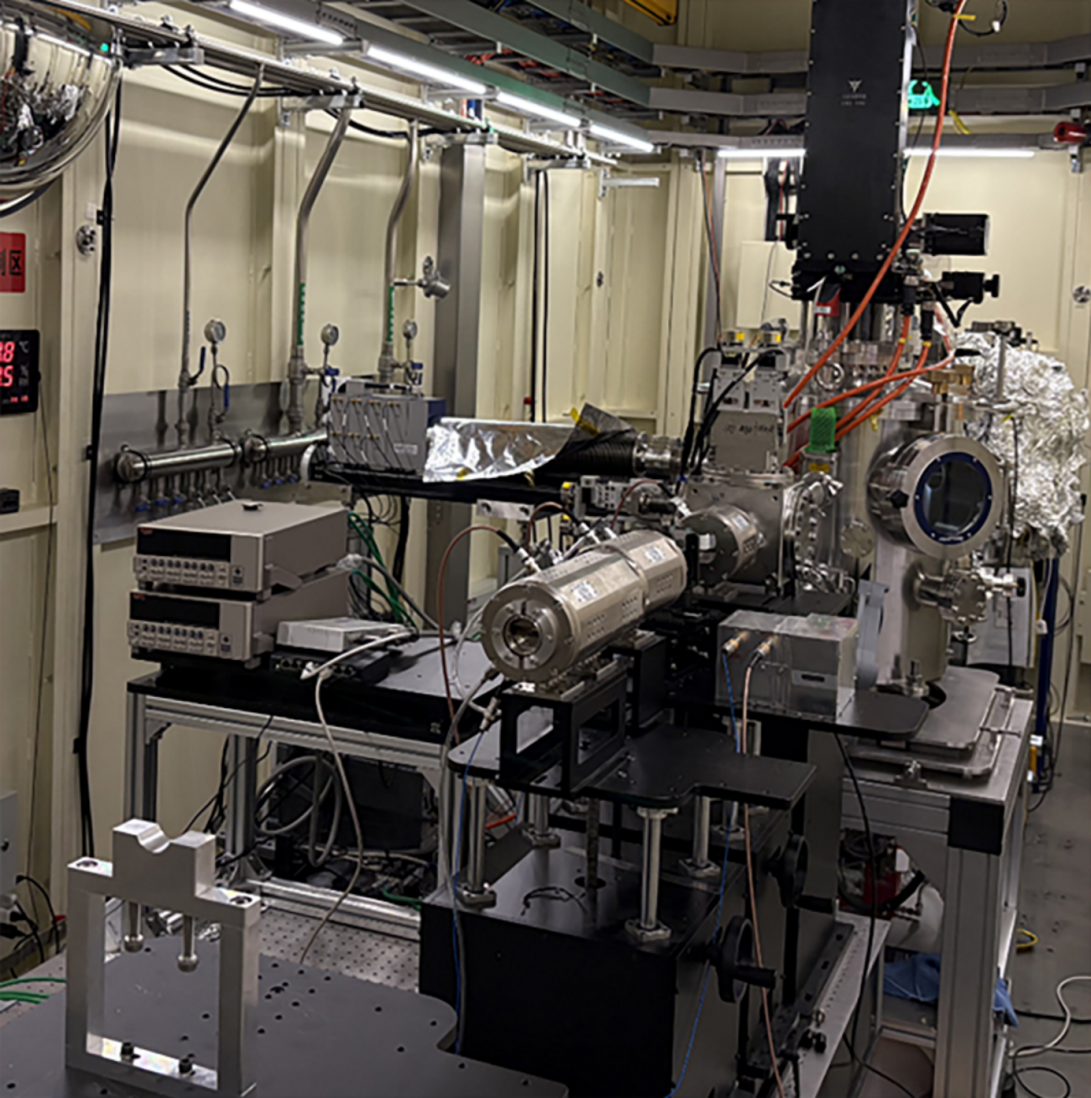
Schematic view of the experimental station.

The layout of the experimental station is given in Fig. 3 ▸. The end-station consists of two parts: a vacuum chamber and the EXAFS setup.

A vacuum chamber is at the focal spot of the TM (at the end of the beamline), and the beam size at the focus spot is 0.127 mm × 0.124 mm (H×V) @ 10 keV. It can achieve a vacuum degree below 1 × 10^−4^ Pa for total electron yield (TEY) and partial fluorescence yield (PFY). The vacuum chamber is manufactured according to standard high vacuum chambers, so it can also achieve a strictly helium-sealed environment. When the pumping valve is closed, and the vacuum chamber is filled with helium at 1 atm pressure, relevant *in situ* experimental research can be carried out. PFY measurements are primarily employed when the mass percentage of the target element in the sample is relatively low (typically <1%). Under such conditions, the TEY method suffers from poor signal-to-noise ratio, making data interpretation challenging. In contrast, the PFY method enables acquisition of high-quality XAFS spectra. It uses a seven-channel silicon drift detector system (SDD; Rayspec) with the effective area of each sensor sensitive region of 30 mm^2^ for the vacuum chamber. Its electronics are Xspress3X.

Downstream of the chamber is a four-blade slit to define the beam size for EXAFS experiments. Downstream of the slit is the EXAFS measurement system, which is located 1.5 m downstream of the focal spot. The beam size in the EXAFS setup is 5 mm × 0.5 mm (H × V). It allows for absorption spectroscopy experiments using the transmission method and fluorescence yield. The transmission method is primarily used for XAFS studies and can be performed under ambient conditions (in air). It houses three ionization chambers (1, 2 and 3) aligned along the beam axis. The SDD detector (Rayspec, UK) and a Lytle ionization chamber (in house) were placed between ionization chamber 1 (AVS, USA) and ionization chamber 2 (AVS, USA).

## 3. Beamline performance

**Figure 4:**
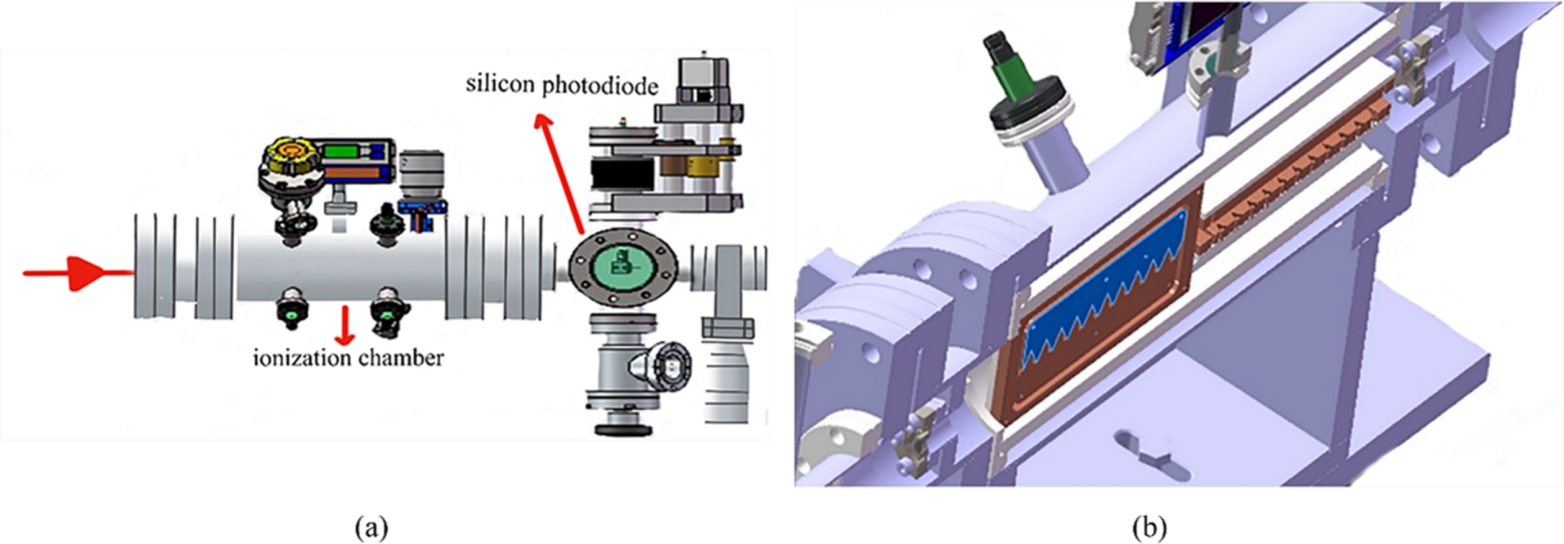
Structure of (*a*) the diagnostic system and (*b*) the negative-pressure ionization chamber.

In order to determine the performance of the beamline, a beamline diagnostic system was designed. This system was located downstream of the HSM and consists of a low-pressure ionization chamber and a silicon photodiode, shown in Fig. 4 ▸, which can realize dynamic monitoring of the beamline.

The low-pressure noble-gas ionization chamber is primarily made up of a chamber, upstream and downstream windows, electrode plates and working gas. The ion chamber is widely used to determine the photon flux (Samson, 1964 ▸) and for XAS (Ho, 1998 ▸), in particular to verify the total instrumental resolution of the beamline (Domke *et al.*, 1992 ▸). The negative-pressure ionization chamber’s electrode is composed of two sawtooth plates that are insulated from each other. It monitors the compensating currents S1 and S2. The relative difference in current, (S1−S2)/(S1+S2), is proportional to the position deviation of the beam, while S1+S2 represents the intensity of the light source. The modified configuration uses 25 µm-thick beryllium windows at both the entrance and exit of the ionization chamber, with adjustable operating pressure maintained between 50 and 5000 Pa. This optimization ensures consistent photon absorption efficiency of 15–20%. Furthermore, by employing two pairs of mutually orthogonal electrode plates, the position deviations of the beam in two directions can be measured, to realize dynamic monitoring of the light intensity signal and position information of the beam (*X* horizontal, *Z* vertical).

The beam passes through the negative-pressure ionization chamber and enters the silicon photodiode. The silicon photodiode can measure the photon flux compared with the negative-pressure ionization chamber. The results of the beamline commissioning are presented below.

### 3.1. Photon energy range

**Figure 5:**
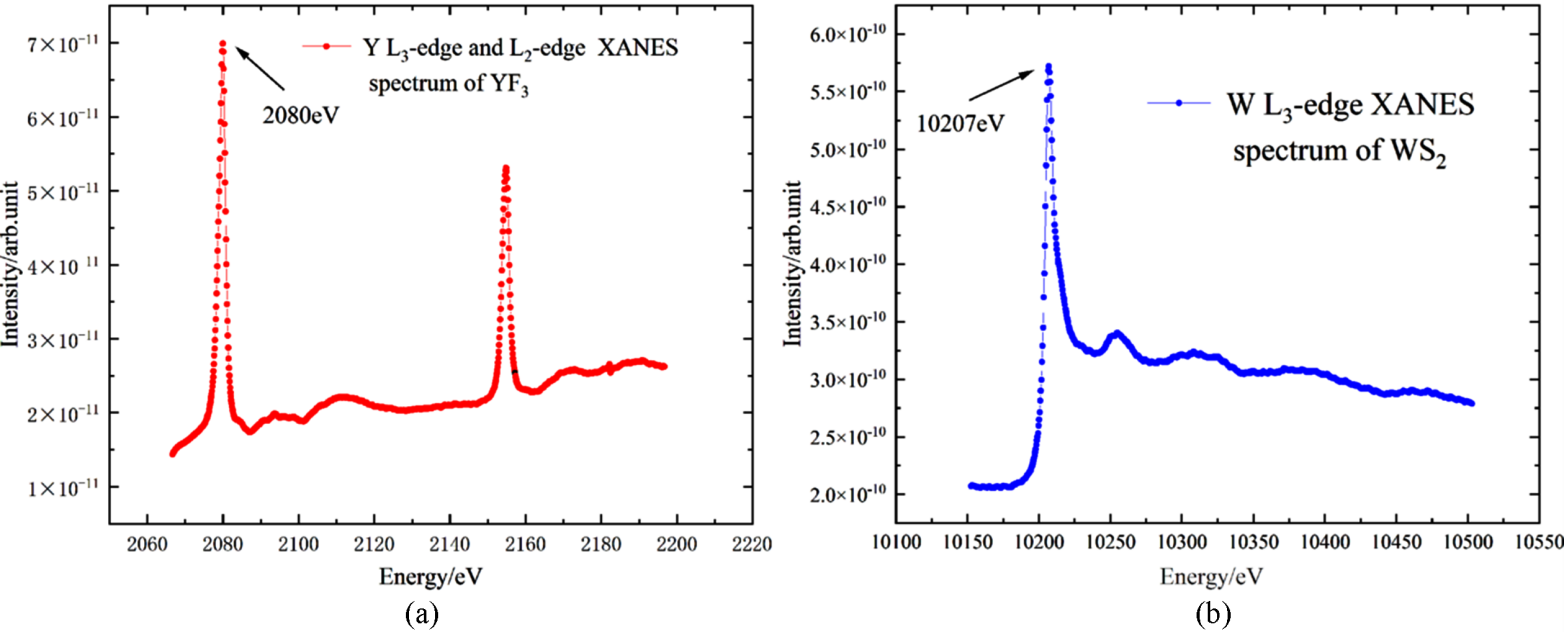
(*a*) Y *L*_3_-edge and *L*_2_-edge XANES spectrum of YF_3_; (*b*) W *L*_3_-edge XANES spectrum of WS_2_.

To verify the energy range (2.1 keV–11 keV), powder samples of YF_3_ and WS_2_ were placed at the sample holder, and TEY spectra were measured for the Y *L*_3_-edge (2080 eV) and the W *L*_3_-edge (10207 eV). The experimental results, shown in Fig. 5 ▸, demonstrate that the energy coverage meets the design requirements.

### 3.2. Energy resolution

Under a given Bragg angle, the resolution of the beamline is typically characterized by the ratio of energy width (*E*/Δ*E*) (Erko *et al.*, 1996 ▸). Essentially, in XAS experiments, the total resolution (Δ*E*_total_) of an element’s absorption edge is limited by both the natural width of the core level (Γ) (Krause & Oliver, 1979 ▸) and the beamline bandwidth (Δ*E*). To characterize the energy resolution of the beamline, it was experimentally verified by measuring absorption spectra of argon and sodium thio­sulfate pentahydrate (Na_2_S_2_O_3_·5H_2_O).

**Figure 6:**
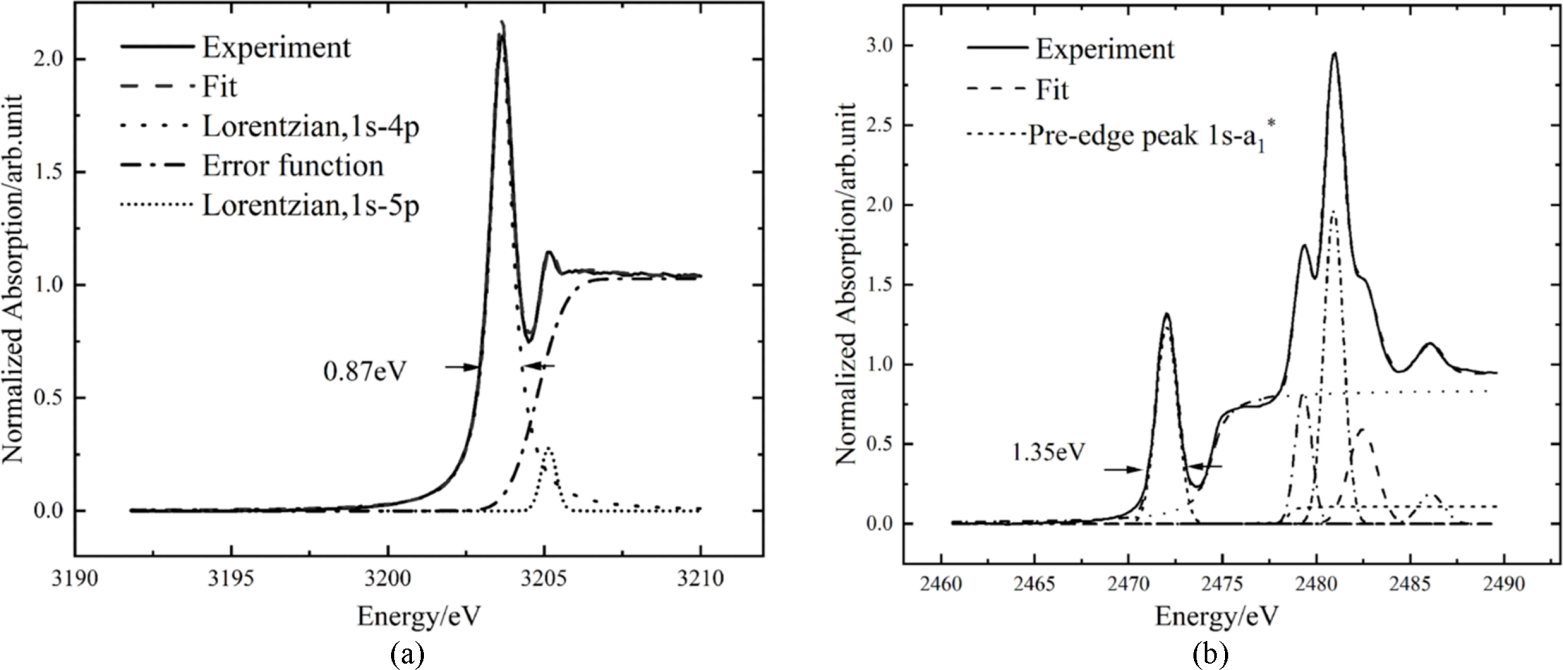
(*a*) Ar *K*-edge XANES spectrum of Ar gas. The fit curve is the sum of two Lorentzian functions and the error function. (*b*) S *K*-edge XANES spectrum of Na_2_S_2_O_3_·5H_2_O.

The absorption spectrum of argon (Lytle *et al.*, 1984 ▸) was measured using an ionization chamber with a pressure of 500 Pa and a step size of 0.1 eV. As shown in Fig. 6 ▸, which shows the white line peak observed in the XANES spectrum of argon at 3203.6 eV, the theoretical width of this peak is the sum of the natural linewidths of the *K* and *N* levels,*i.e.* Γ_*K*_ + Γ_*N*_. Since the natural linewidth Γ_*N*_ of argon gas is much smaller than Γ_*K*_ (0.68 eV), it can be neglected (Krause & Oliver, 1979 ▸). Thus, the theoretical width of the white line peak for argon gas is 0.68 eV. However, the experimentally measured white line peak width (Δ*E*_total_) is 0.87 eV, which is significantly larger than the theoretical width. This discrepancy is due beamline broadening. According to the formula
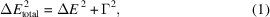
the beamline bandwidth Δ*E* can be calculated to be 0.54 eV. Consequently, the resolution of the beamline at 3203.6 eV is approximately 5904.

Similarly, the TEY absorption spectrum of Na_2_S_2_O_3_·5H_2_O was measured. As shown in Fig. 6 ▸, the theoretical width of the pre-edge peak (2472 eV) is 1.29 eV (Song *et al.*, 1995 ▸). The experimentally measured total broadening (Δ*E*_total_) is 1.35 eV. According to formula (1), the instrumental broadening of the beam can be calculated to be 0.397 eV. This corresponds to an energy resolution of 6227 at 2472 eV.

**Figure 7:**
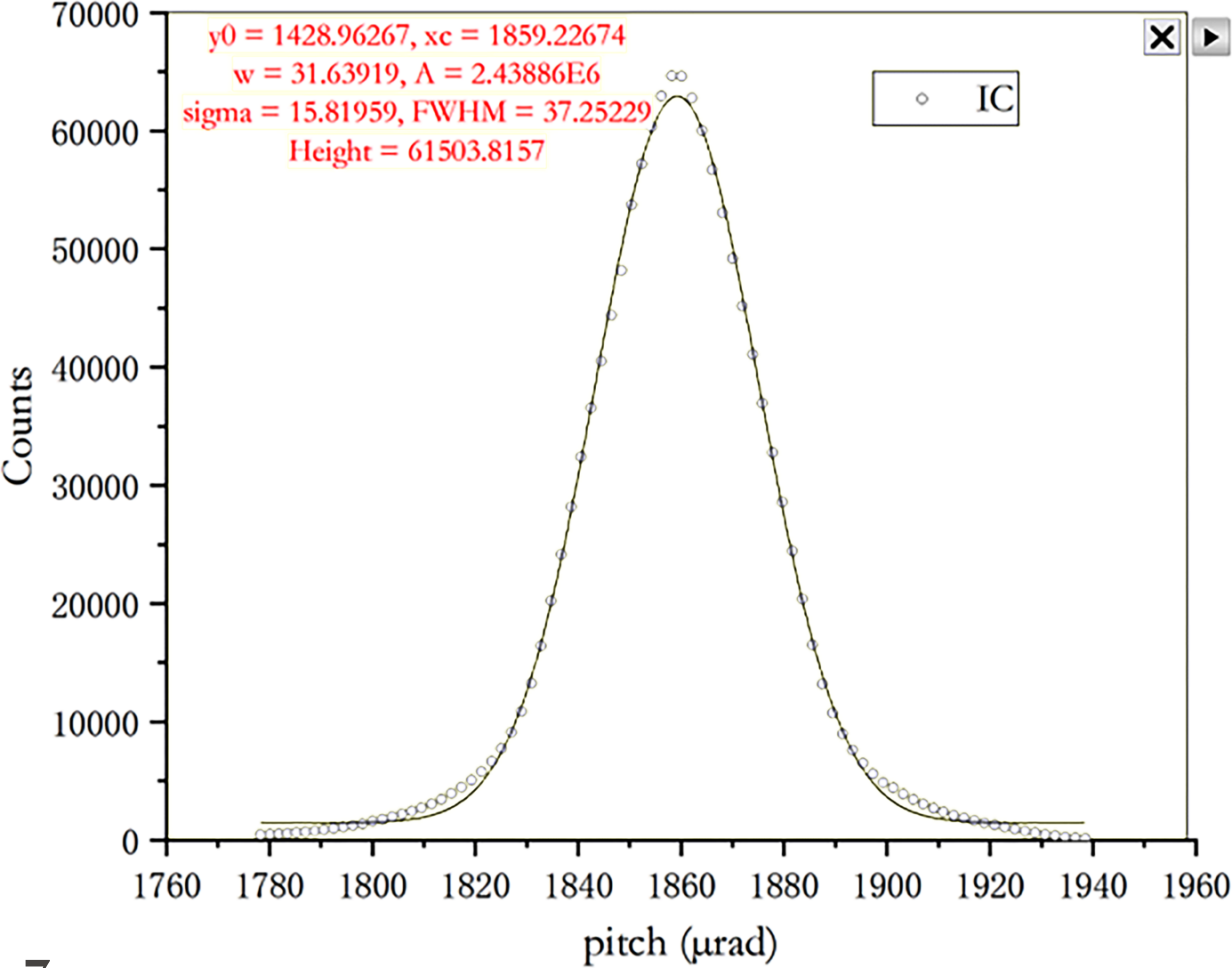
Rocking curve of the monochromator at 10 keV.

A rocking curve of 10 keV was measured through the second crystal, and the measured full width at half-maximum was 37.3 µrad, which was basically consistent with the theoretical value (38 µrad), as shown in the Fig. 7 ▸. This is just the monochromator’s performance. The resolution of the entire beamline is measured using argon and sodium thio­sulfate pentahydrate (Na_2_S_2_O_3_·5H_2_O), which can be affected by the heat load—especially at lower energies where the power density increases and thermal effects become more significant. Across the full energy range there is a compromise between resolution, focal spot and flux, although theoretically these metrics can be achieved at the same time.

### 3.3. Photon flux

**Figure 8:**
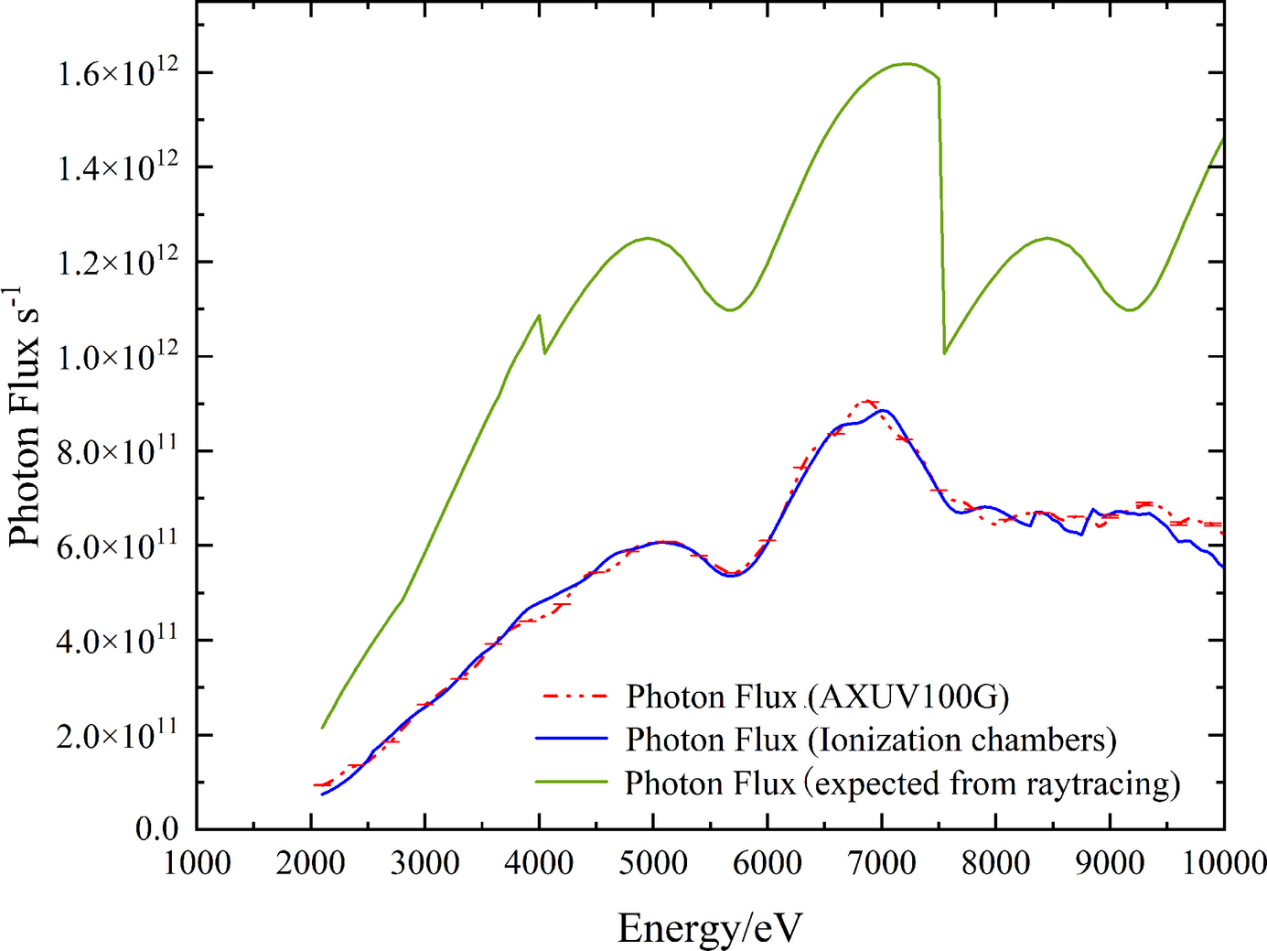
Measured photon flux by silicon photodiode and ionization chamber. The measurements differ by about 5%.

There is currently no suitable device available for directly counting the high flux of X-ray photons produced by synchrotron radiation sources (Owen *et al.*, 2009 ▸). Ionization chambers are commonly used devices for measuring photon flux on synchrotron beamlines (Wyckof, 1979 ▸; Nariyama, 2006 ▸). The relationship between the current *I* = *S*1+*S*2 and photon flux is given by the following equation,
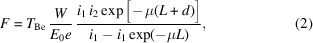

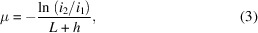
where *F* is the photon flux, *i*_1_ is the current of the upstream ionization chamber, *i*_2_ is the current of the downstream ionization chamber, *W* is the average ionization energy of the gas (24.3 eV), *T*_Be_ is the transmission of the window (Be), *e* is the elementary charge, *E*_0_ is the incident photon energy, *d* is the length of the ionization chamber’s protecting electrode, μ is the linear absorption coefficient of the material, *h* is the gap between the collection electrode of the ionization chamber and *L* is the length of the ionization chamber’s collection electrode. During the experiment, krypton gas was introduced into the ionization chamber at a specific pressure to maintain the photon absorption by krypton at 15–20%. The measured photon flux curve at the sample position is shown in Fig. 8 ▸ (converted to storage ring current 200 mA).

In addition to ionization chambers, silicon photodiodes (AXUV-100G from Opto Diode) are also commonly used devices for flux measurement (Jach & Cowan, 1983 ▸; Krumrey *et al.*, 2006 ▸; Alkire & Rotella, 1997 ▸), offering higher accuracy compared with ionization chambers. The separated electrons and holes form a photocurrent. The relationship between the photocurrent and photon flux is given by the following equation,
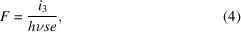
where *F* is the photon flux, *i*_3_ is the measured current, *s* is the sensitivity of the photodiode calibrated in the PTB laboratory, *e* is the elementary charge, and *h*ν is the photon energy.

In the experiment, the silicon photodiode placed at the sample position fully receives monochromatic light, and the photocurrent curve was measured using a Keithley 6517B electrometer. The corresponding photon flux curve was then derived, as shown in Fig. 8 ▸.

Due to unstable beam current in the early stage of the storage ring construction, the flux measurement value has been normalized to the equivalent result for a 200 mA beam current. The measurement results indicate that the photon flux reaches 9 × 10^11^ photons s^−1^ @ 200 mA @ 6 GeV. The curves from measurement and ray-tracing differ by 45%. This difference is primarily attributed to double-crystal parallelism and carbon contamination. It also includes the roughness from each mirror (CM, TM and HSM) and the deviation between the actual incident angle of the mirror and the design value. The measurement curves obtained from the two methods differ by approximately 5%. This difference is primarily attributed to inaccuracies in the purity and pressure of the gas inside the ionization chamber. Although the ionization chamber has a larger measurement uncertainty compared with diodes when measuring flux, it remains advantageous due to its capacity for dynamic measurement. There are special fluctuations in both the expected and measured flux, which are due to switching of the HSM coating and the reflectivity bilayer coating B_4_C/Pt (CM and TM).

### 3.4. Stability

**Figure 9:**
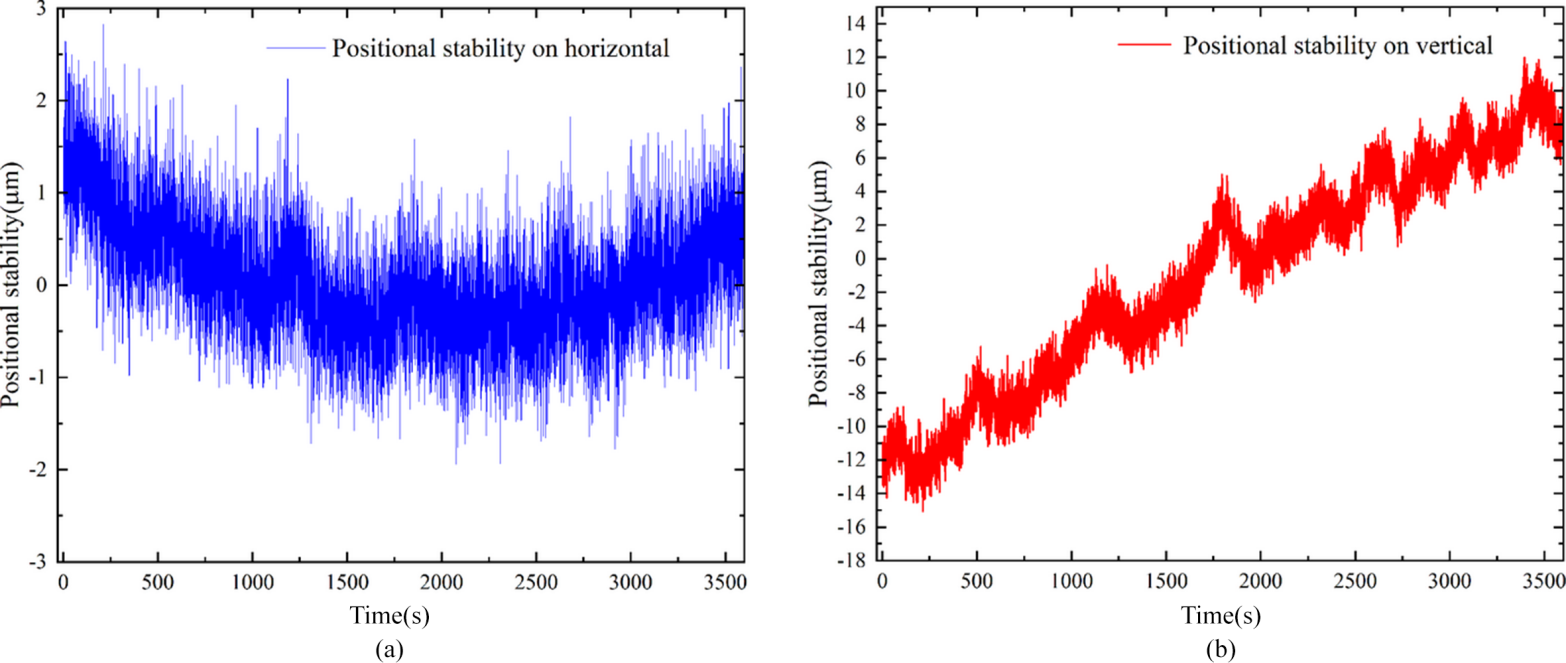
Position stability of light spots in (*a*) the horizontal directions and (*b*) the vertical directions.

The stability of the beam position is measured using the negative-pressure ionization chamber. First, the vertical direction response of the ionization chamber is measured by adjusting the pitch angle of the DCM, and similarly by adjusting the roll angle to measure the responsiveness in the horizontal direction. Based on the responsiveness of the ionization chamber and the distance from the monochromator to the focal spot, the position change of the focal spot can be further calculated,
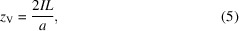

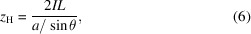

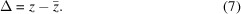
*a* (or *a*/sinθ) is the ionization chamber signal variation in the vertical (or horizontal) direction per 1 µrad change in pitch (or roll) angle, θ is the Bragg angle, *I* = (*S*1−*S*2)/(*S*1+*S*2) is the ionization chamber reading, *L* is the distance from the monochromator to the focal spot, *z* is the focal spot position and Δ is the position change of the focal spot. The results, presented in Fig. 9 ▸, show that, for every 1 µrad change of the pitch (microradian), the ionization chamber reading changes by 0.0059. In the horizontal direction, the ionization chamber reading change is 0.0053 for every 1 µrad of the roll change. In this diagnostic measurement, the stability in the horizontal and vertical directions was assessed over a period of 3600 s using the ionization chamber. The fluctuation of the focal spot position in the horizontal direction did not exceed ±3 µm @ 7 keV, while in the vertical direction it did not exceed ±15 µm @ 7 keV. The light position fluctuation is small for a focus spot of size 127 µm × 124 µm (H × V).

**Figure 10:**
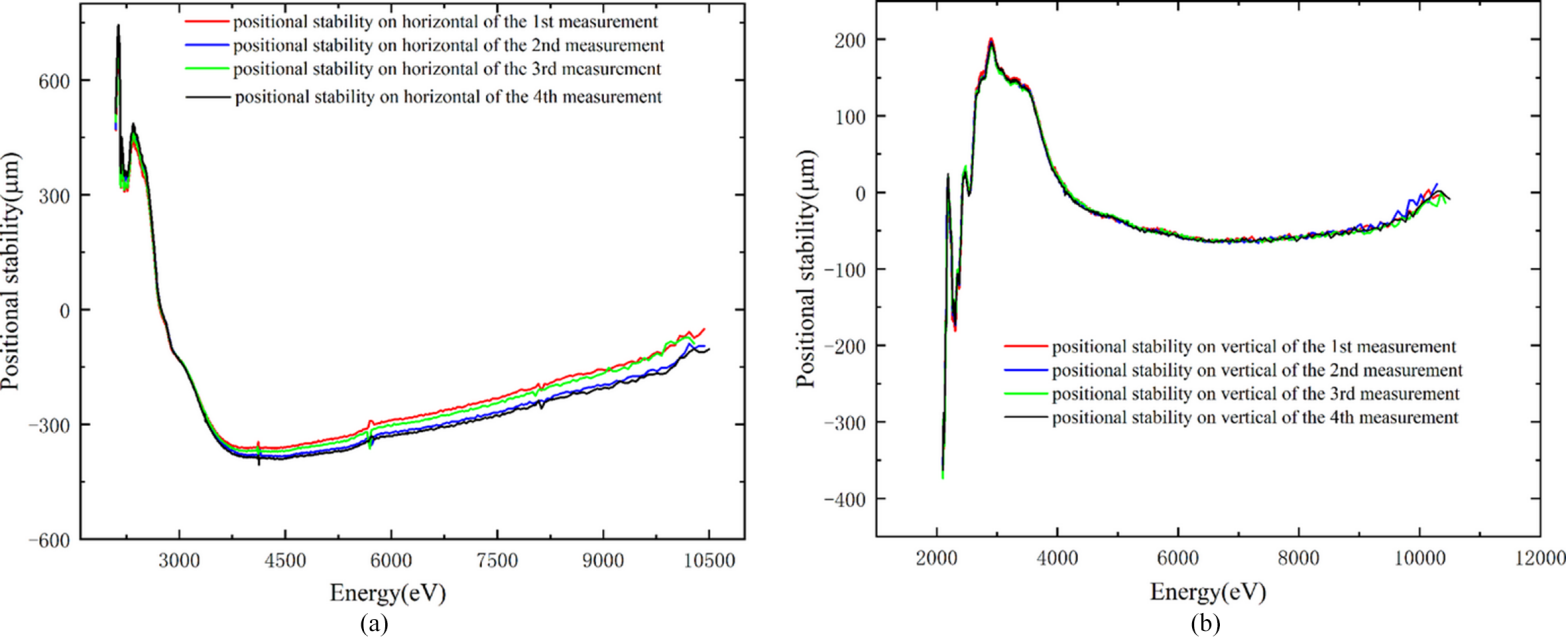
Scanning stability of the DCM; each direction is repeated four times. Position stability of light spots in (*a*) the horizontal directions, and (*b*) the vertical directions.

The longer term and scanning stability of the monochromator have been measured, and are given in Fig. 10 ▸. The beam spot shifts by ±600 µm × ±200 µm (H × V) at 2.1–11 keV. The deviation is significant at low energies and smaller at high energies. For example, within the 1000 eV range beyond the Fe *K*-edge, the shift is only 30 µm × 5 µm (H × V). Therefore, the beamline remains suitable for EXAFS.

**Figure 11:**
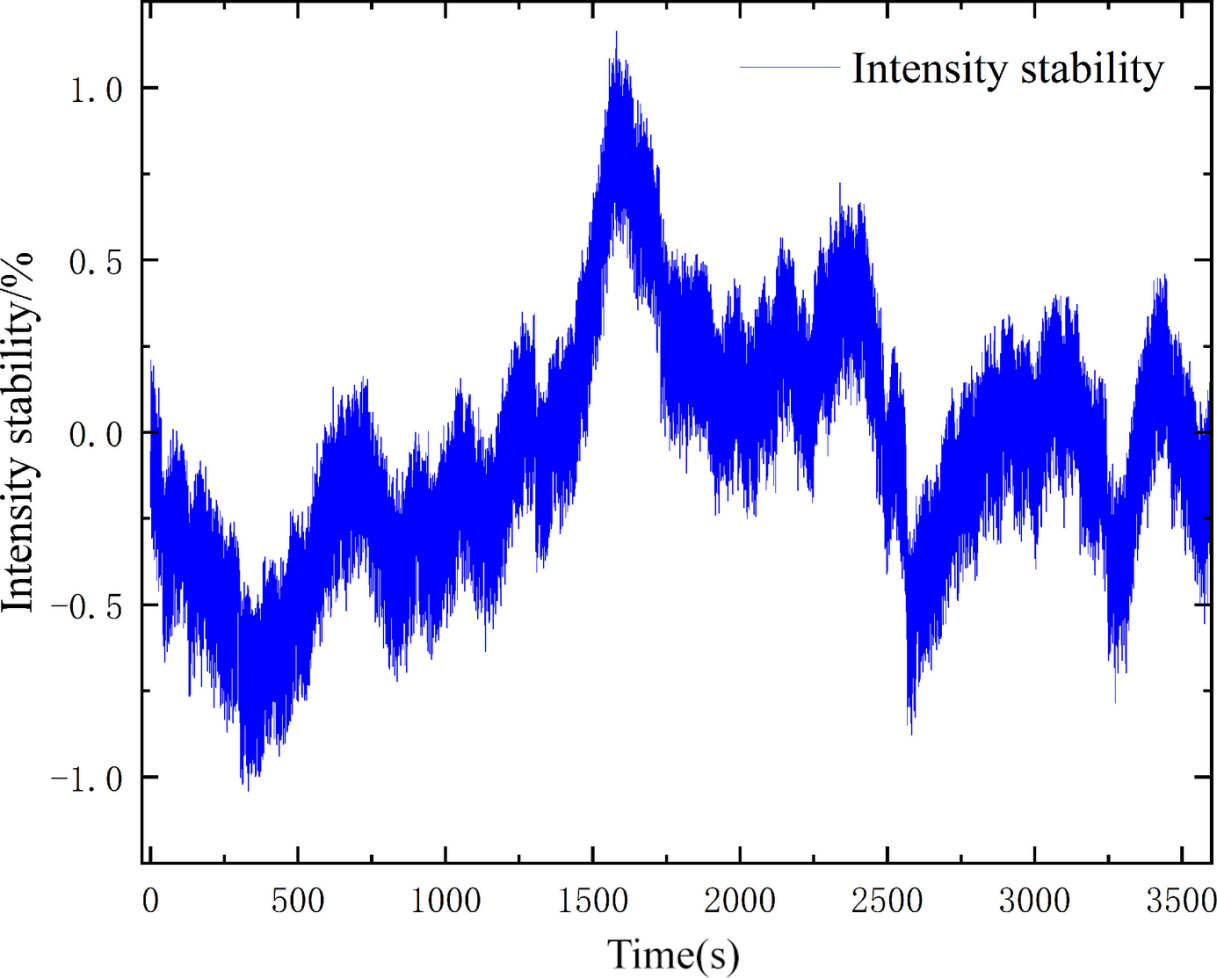
The fluctuation of the intensity.

The intensity stability was also monitored over a long time, and is given in Fig. 11 ▸. The fluctuation of the intensity did not exceed 1.2% @ 7 keV for every hour.

### 3.5. Higher-order harmonics

Although a wide energy range is a very desirable feature of synchrotron radiation it has one main drawback: the contamination of the photon beam by higher-order harmonics. Diagnosis of higher-order harmonics is mainly divided into two methods: transmission gratings and filtering.

Transmission gratings are always employed for soft X-rays. For tender and hard X-rays, transmission gratings demand more stringent requirements for both thickness and linewidth, which are challenging to achieve with current fabrication techniques. Filtering is based on the differential in transmission rates of the fundamental wave and its harmonics through various media. When the light passes through a specific medium, the fundamental wave may be completely absorbed, while the harmonics can partially or fully transmit through. This selective transmission allows the determination of the ratio between the fundamental wave and the harmonics,
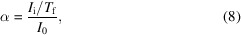
where *I*_0_ is the signal of the incident light without filtering, *I*_i_ is the signal of incident light with filtering and *T*_f_ is the transmission for higher-order harmonics of filtering. There is no second harmonics in the beamline, because of the Si (111) crystals. The beamline energy is up to 11 keV, there are almost no fifth harmonics, mainly third and fourth harmonics, and the harmonics mainly exist between 2.1 and 3.5 keV.

**Figure 12:**
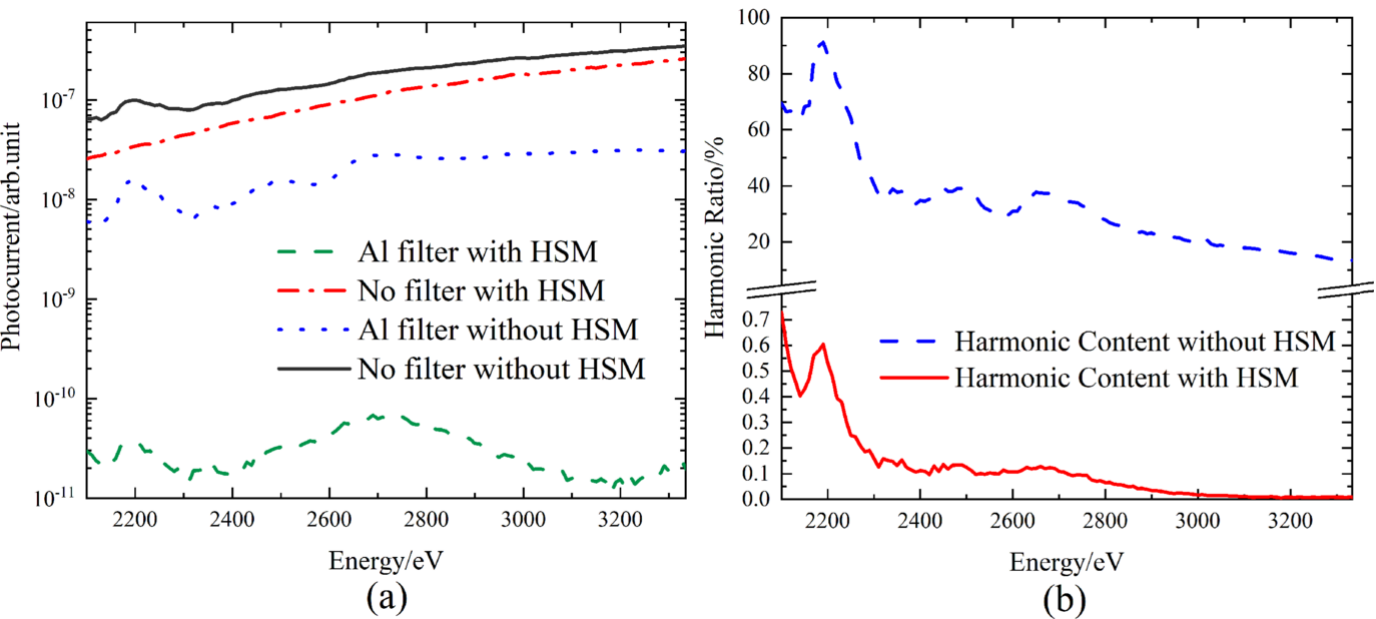
(*a*) Photocurrent response before and after adding the filter. (*b*) Harmonic ratio before and after adding the harmonic suppression mirror; when the HSM was introduced into the beamline, the harmonic ratio was reduced to below 0.7%.

In this experiment, the filtering method was primarily employed. An 80 µm Al filter foil was used to measure the harmonic curves both with and without an HSM in the beamline. The HSM contains three layers of coatings (B_4_C, Ni and Pt) with working energy ranges corresponding to 2.1 keV–4.4 keV, 4.0 keV–7.8 keV and 7.5 keV–11 keV, and 7 mrad of the incident angle. The experimental results (Fig. 12 ▸) show that, when the HSM was introduced into the beamline, the harmonic ratio was reduced to lower than 0.7%–0.009% (2100 eV–3350 eV).

**Figure 13:**
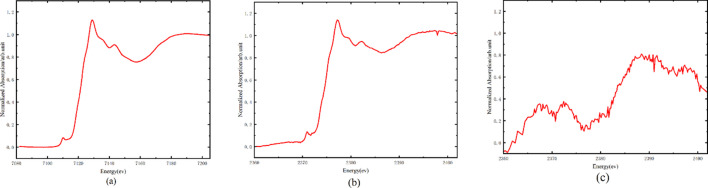
(*a*) Fe *K*-edge absorption spectra (TEY) acquired from 7080 to 7200 eV; (*b*) Fe *K*-edge absorption spectra (TEY) acquired from 2360 to 2400 eV without the harmonic suppression mirror; (*c*) Fe *K*-edge absorption spectra (TEY) acquired from 2360 to 2400 eV with the harmonic suppression mirror.

In order to more visually observe the ratio of harmonics, Fe foil could be used to look at harmonics near 2370 eV by looking for an edge step when scanning through this energy. Fe foil has been scanned across two energy ranges: 7080–7200 eV and 2360–2400 eV (Fig. 13 ▸).

### 3.6. Spot size

**Figure 14:**
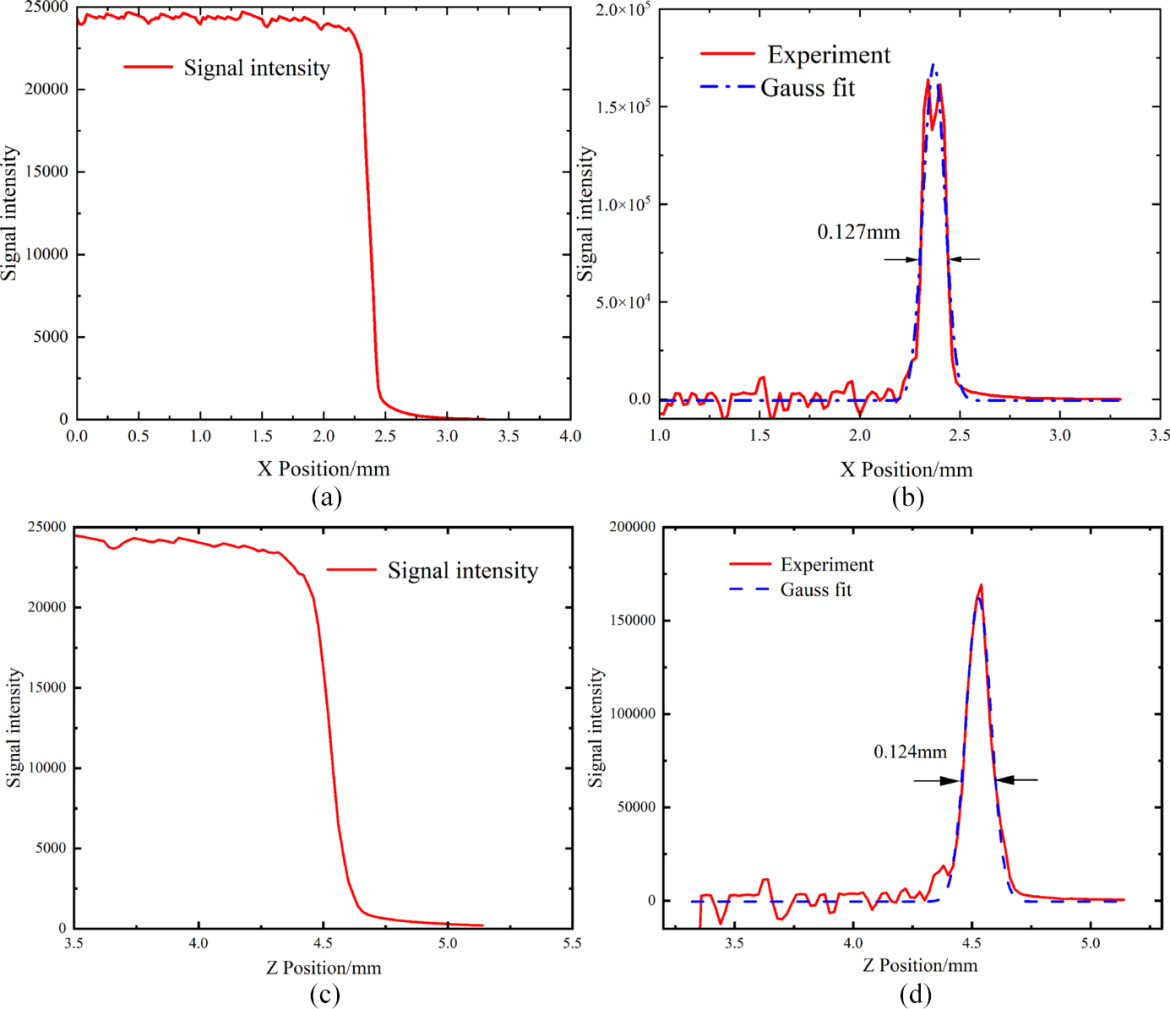
(*a*) Signal intensity of the tungsten blade in the horizontal. (*b*) Spot size in the horizontal. (*c*) Signal intensity of the tungsten blade in the vertical. (*d*) Spot size in the vertical.

In the TEX beamline, the sample stage is precisely positioned at the focal point of the beam. To accurately measure the beam spot size, tungsten blades are used. Two tungsten blades were placed orthogonally on the sample stage. Results of the scanning process of the blades are shown in Fig. 14 ▸, where the focal spot was scanned in both the horizontal and vertical directions, and the results were differentiated. The final measured focal spot size was 0.127 mm × 0.124 mm (H × V) @ 10 keV.

## 4. XAFS results

**Figure 15:**
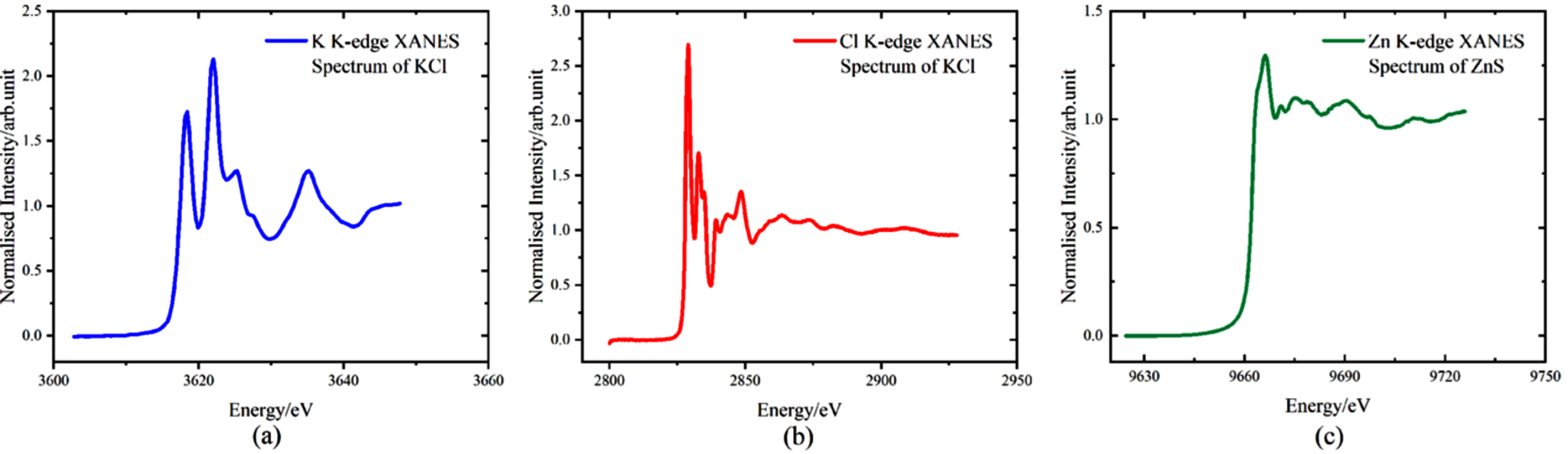
K *K*-edge (*a*) and Cl *K*-edge (*b*) XANES spectrum (TEY); (*c*) Zn *K*-edge XANES spectrum (PFY).

Three experimental modes are available: TEY, PFY and transmission. Absorption spectra were measured using all three methods (Figs. 15 ▸ and 16 ▸).

The advantage of the TEY method lies in its sensitivity to surface information. It can characterize a surface layer approximately 5 nm thick, making it suitable for studying surface or interfacial elemental information. However, this method requires the sample to be conductive.

The fluorescence yield method is suitable for samples with low concentrations of the target element and can be used for *in situ* experiments.

**Figure 16:**
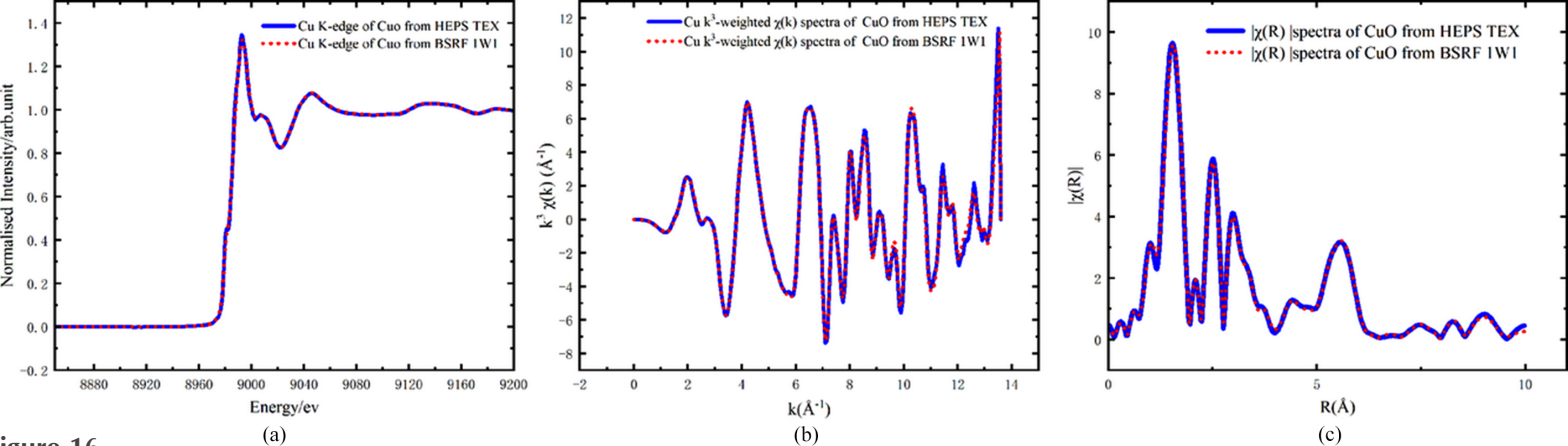
(*a*) Cu *K*-edge XANES spectrum (transmission). (*b*) Cu *k*^3^-weighted χ(*k*) spectra. (*c*) Magnitude of Fourier-transformed χ(*R*) spectra.

The transmission method is the most commonly used in XAS and can be carried out directly in the atmosphere. The transmission method requires higher homogeneity of the sample. Fig. 16 ▸ shows the *K*-edge absorption spectrum of Cu in CuO (Aladdin) powder recorded at the BSRF 1W1B station in comparison with our data. Fourier transformation was carried out using the *Athena* software (Ravel & Newville, 2005 ▸). The measured spectra demonstrate that the TEX beamline possesses the capability to support XAFS experiments.

## 5. Conclusion

This paper introduces the design index and diagnostic results of the TEX beamline of HEPS and its experimental station. The energy resolution reaches 6000, and the photon flux at the sample point is up to 9 × 10^11^ photons s^−1^ @ 6900 eV. Harmonics are greatly reduced by the harmonic suppression mirror, which enables the experiment to measure the *K*-edge XANES of sulfur and phospho­rus. The position instability of the beamline is controlled below ±3 µm × 15 µm (H × V), which makes the experimental data more reliable. Through testing of three kinds of absorption spectrum experiment methods, it is proved that the end-station is reliable, simple and flexible, and covers a wide range of applications. TEX is the first bending magnet beamline of HEPS. Its diagnosis and commissioning are of great significance for the construction of subsequent bending magnet beamlines of HEPS.

## References

[bb2] Alkire, R. W. & Rotella, F. J. (1997). *J. Appl. Cryst.* **30**, 327–332.

[bb3] Chantler, C. T., Bunker, G., D’Angelo, P. & Diaz-Moreno, S. (2024). *Nat. Rev. Methods Primers* **4**, 89.

[bb4] Domke, M., Mandel, T., Puschmann, A., Xue, C., Shirley, D. A., Kaindl, G., Petersen, H. & Kuske, P. (1992). *Rev. Sci. Instrum.* **63**, 80–89.

[bb5] Erko, A. I., Schäfers, F., Gudat, W. I., Abrosimov, N., Rossolenko, S. N., Alex, V., Groth, S. & Schröder, W. (1996). *Nucl. Instrum. Methods Phys. Res. A* **374**, 408–412.

[bb7] Grünert, W. & Klementiev, K. (2020). *Phys. Sci. Rev.* **5**, 20170181.

[bb8] Ho, G. H. (1998). *Chem. Phys.* **226**, 101–111.

[bb9] Jach, T. & Cowan, P. L. (1983). *Nucl. Instrum. Methods Phys. Res.* **208**, 423–425.

[bb10] Jiao, Y., Bai, Z. H. & Li, X. (2022). *High Power Laser Particle Beams* **34**, 104004.

[bb11] Jiao, Y. & Pan, W. M. (2022). *High Power Laser Particle Beams* **34**, 104002.

[bb12] Krause, M. O. & Oliver, J. H. (1979). *J. Phys. Chem. Ref. Data* **8**, 329–338.

[bb13] Krumrey, M., Gerlach, M., Scholze, F. & Ulm, G. (2006). *Nucl. Instrum. Methods Phys. Res. A* **568**, 364–368.

[bb14] Lytle, F. W., Greegor, R. B., Sandstrom, D. R., Marques, E. C., Wong, J., Spiro, C. L., Huffman, G. P. & Huggins, F. E. (1984). *Nucl. Instrum. Methods Phys. Res. A* **226**, 542–548.

[bb15] Nariyama, N. (2006). *Phys. Med. Biol.* **51**, 5199–5209. 10.1088/0031-9155/51/20/008 17019033

[bb16] Okui, M., Shimoguchi, A., Yato, N., Murayama, N., Kikuchi, I., Tsuboki, I. & Kanda, K. (2022). *J. Phys. Conf. Ser.* **2380**, 012054.

[bb17] Owen, R. L., Holton, J. M., Schulze-Briese, C. & Garman, E. F. (2009). *J. Synchrotron Rad.* **16**, 143–151. 10.1107/S0909049508040429 PMC2651761 19240326

[bb100] Ravel, B. & Newville, M. (2005). *J. Synchrotron Rad.* **12**, 537–541. 10.1107/S0909049505012719 15968136

[bb18] Samson, J. A. R. (1964). *J. Opt. Soc. Am.* **54**, 6–15.

[bb22] Song, I., Rickett, B. I., Janavicius, P. V., Payer, J. H. & Antonio, M. R. (1995). *Nucl. Instrum. Methods Phys. Res. A* **360**, 634–641.

[bb23] Sun, Z. & Li, M. (2024). *Physics* **53**, 80–88.

[bb25] Wyckof, H. O. (1979). Editor. *Average Energy Required to Produce an Ion Pair.* ICRU Report 31. International Commission on Radiation Units and Measurements, Bethesda, MD 20814-3095, USA.

